# Sex-differences in LPS-induced neonatal lung injury

**DOI:** 10.1038/s41598-019-44955-0

**Published:** 2019-06-11

**Authors:** Leanna Nguyen, Odalis Castro, Robyn De Dios, Jeryl Sandoval, Sarah McKenna, Clyde J. Wright

**Affiliations:** Section of Neonatology, Department of Pediatrics, University of Colorado School of Medicine, Aurora, CO 80045 USA

**Keywords:** Toll-like receptors, Respiratory distress syndrome

## Abstract

Being of the male sex has been identified as a risk factor for multiple morbidities associated with preterm birth, including bronchopulmonary dysplasia (BPD). Exposure to inflammatory stress is a well-recognized risk factor for developing BPD. Whether there is a sex difference in pulmonary innate immune TLR4 signaling, lung injury and subsequent abnormal lung development is unknown. Neonatal (P0) male and female mice (ICR) were exposed to systemic LPS (5 mg/kg, IP) and innate immune signaling, and the transcriptional response were assessed (1 and 5 hours), along with lung development (P7). Male and female mice demonstrated a similar degree of impaired lung development with decreased radial alveolar counts, increased surface area, increased airspace area and increased mean linear intercept. We found no differences between male and female mice in the baseline pulmonary expression of key components of TLR4-NFκB signaling, or in the LPS-induced pulmonary expression of key mediators of neonatal lung injury. Finally, we found no difference in the kinetics of LPS-induced pulmonary NFκB activation between male and female mice. Together, these data support the conclusion that the innate immune response to early postnatal LPS exposure and resulting pulmonary sequelae is similar in male and female mice.

## Introduction

A growing body of literature supports the hypothesis that many morbidities associated with prematurity are sex-specific^1^. An increased risk of respiratory morbidity, including bronchopulmonary dysplasia (BPD), has been noted in preterm males^2–4^. Although genetic, developmental and hormonal differences between male and females have been identified, the mechanisms underlying the increased respiratory morbidity observed in preterm male infants are unclear.

The pathogenesis of BPD is multifactorial. Both clinical and pre-clinical data support a role played by oxidant and inflammatory stress in mediating neonatal lung injury and subsequent abnormal development^5–10^. Of note, pre-clinical data have begun to unravel the mechanisms underlying sex-type specific responses to neonatal hyperoxia exposure. Compared to female mice, neonatal male mice were more susceptible to early postnatal hyperoxia-induced lung injury and abnormal lung development^11^. This injury was associated with markers of differential NFκB activity between male and female mice. Work from this same group demonstrated evidence of increased hyperoxia-induced NFκB activity in human umbilical vein endothelial cells derived from female donors^12^. These data are consistent with previous reports demonstrating that in response to hyperoxia, NFκB activity is protective in the neonatal lung^13–16^.

Exposure to inflammatory stress injures the developing lung which results in abnormal development. Stimulating the fetal innate immune response with an intraamniotic (IA) injection of LPS during the saccular stage of lung development induces pulmonary inflammation and impairs alveolarization in fetal sheep and rats^17–25^. In mice, IA LPS during the pseudoglandular (e15) stage of lung development induces inflammation and inhibits distal branching when assessed 48 hours later, and this finding is attenuated by inhibiting the innate immune response^26–29^, while postnatal LPS exposure during the alveolar stage (PN5) of lung development inhibits alveolarization^30,31^. However, whether this inflammatory-stress induced injury is sex-specific is unknown.

Previous studies have demonstrated that NFκB signaling protects the neonatal lung against both oxidant and inflammatory stress-induced injury^16,31^. As stated, pulmonary NFκB activity has been implicated in the attenuated hyperoxia-induced lung injury observed in neonatal female mice and oxygen toxicity observed in HUVEC derived from females^11,12^. However, these findings may not be applicable to inflammatory stress-induced models of lung injury. The signaling mechanisms leading to NFκB activation following exposure to inflammatory stimuli are distinct from those observed following exposure to oxidant stress. In quiescent cells, NFκB remains sequestered in the cytoplasm bound to members of the IκB family of inhibitory proteins^32^. Following inflammatory stress, IκB phosphorylation and degradation allow NFκB nuclear translocation and DNA binding^33^. In contrast, the well-defined NFκB activation cascade that occurs after exposure to inflammatory stress, a definitive pathway has not been established following exposure to oxidant stress^34–37^. Thus, any sex-type specific differences in response to hyperoxia may not be applicable to injuries following exposure to inflammatory stress.

In this study, we sought to determine whether LPS-induced neonatal lung injury was sex-type specific, and to evaluate the hepatic and pulmonary innate immune response to LPS challenge in the immediate postnatal period. We report that there are no differences in pulmonary injury and abnormal lung development between male and female mice exposed to early postnatal systemic LPS challenge. Furthermore, we demonstrate that pulmonary expression of factors previously implicated in sex differences and lung injury, as well as the key components of TLR4-NFκB signaling are similar in neonatal male and female mice. Together, these data support the conclusion that the innate immune response to early neonatal LPS exposure and the resulting pulmonary sequelae are similar in male and female mice.

## Methods

### Murine model of endotoxemia

Neonatal (P0, male and female) ICR mice were exposed to LPS (Sigma L2630, 5 mg/kg, IP). The litter was split in half into control and exposed groups. At this point, the best visual determination of sex made in an attempt to equally distribute males and females to control and exposed groups. Sex was confirmed by testing for SRY by qPCR on mRNA isolated from hepatic tissue (see below). All procedures were approved by the IACUC at the University of Colorado (Aurora, CO) and care and handling of the animals was in accord with the National Institutes of Health guidelines for ethical animal treatment.

### Lung inflation and collection of pulmonary tissue

For pulmonary mRNA and protein analysis, P0 mice were sacrificed at 1 or 5 hours of LPS exposure with a fatal dose of pentobarbital sodium. Lungs were perfused with normal saline, removed, snap-frozen, and stored at −80 C. For morphometric assessments, mice were exposed to LPS on P0 and sacrificed on P7 and p28 with a fatal dose of pentobarbital sodium. Following perfusion of the lungs with normal saline, the trachea was cannulated with a 24 G angiocath and the lungs were inflation-fixed at 25 cm H_2_O pressure for 10 minutes with 4% paraformaldehyde. Lungs were paraffin-embedded, and sections were cut (5 µm) and stained with hematoxylin and eosin at the University of Colorado Denver Morphology and Phenotyping Core.

### Morphometric analysis

Radial alveolar counts (RAC), and objective measure of alveolar number, were assessed as previously described^38,39^. Counts were performed on four separate male and female ICR mice for each condition (room air and endotoxemia exposure). The average RAC was obtained from a minimum of 30 perpendicular lines obtained from photomicrographs of 5–10 high-powered fields of two separate sections of lung per animal. Measurements of mean linear intercept (MLI), a measurement of the mean distance in the air spaces, and airspace area (ASA) were performed using the computer-assisted image-analysis program Metamorph Basic (Molecular Devices, Sunnyvale, CA) with custom-designed macros on images captured on an Olympus IX83 microscope (10x, 20x, and 40x objective) and quantified with Metamorph Basic (MolecularDevices Sunnyvale, CA). Ten randomly selected non-overlapping sections per mouse at 40X magnification were assessed.

### Isolation of mRNA, cDNA synthesis and analysis of relative mRNA levels by RT-qPCR

Frozen tissue was placed in RLT buffer (Qiagen) and tissue was homogenized using the Bullet Blender (NextAdvance). Pulmonary and hepatic mRNA was collected from homogenized tissue using the RNeasy Mini Kit (Qiagen) according to the manufacturer’s instructions. Initially, tissue RNA was assessed for purity and concentration using the NanoDrop (ThermoFisher Scientific), and cDNA synthesized using the Verso cDNA synthesis Kit (ThermoFisher Scientific). Relative mRNA levels were evaluated by quantitative real-time PCR using exon spanning primers (Table 1), TaqMan gene expression and StepOnePlus Real-Time PCR System (Applied Biosystems). Relative quantitation was performed via normalization to the endogenous control 18S using the cycle threshold (ΔΔCt) method.

**Table 1:** List of genes for qPCR analysis.

Target	Assay ID
*Bcl2a1*	Mm03646861_mH
*Bcl2l1*	Mm00437783_m1
*Birc3*	Mm01168413_m1
*Ccl2*	Mm00441242_m1
*Cxcl1*	Mm04207460_m1
*Cxcl2*	Mm00436450_m1
*Cxcl10*	Mm00445235_m1
*Ikbkb*	Mm01222247_m1
*Il1a*	Mm00439620_m1
*Il1b*	Mm01336189_m1
*Il6*	Mm00446190_m1
*Il10*	Mm00439614_m1
*Il12b*	Mm00434174_m1
*Myd88*	Mm00440338_m1
*Nfkb1*	Mm00476361_m1
*Nfkbia*	Mm00477798_m1
*Nfkbib*	Mm00456849_m1
*Rel*	Mm00485657_m1
*Rela*	Mm00501346_m1
*Serpinb2*	Mm00440905_m1
*Sry*	Mm00441712_s1
*Ticam1*	Mm00844508_s1
*Ticam2*	Mm01260003_m1
*Tlr4*	Mm00445273_m1
*Tnf*	Mm00443258_m1
*Xiap*	Mm01311594_mH

### Isolation of protein and western blot analysis

Frozen pulmonary tissue was homogenized using the Bullet Blender (NextAdvance). Pulmonary whole cell lysates were collected in T-PER (ThermoFisher Scientific) and cytosolic and nuclear extracts were collected in NE-PER (ThermoFisher Scientific). Lysates, cytosolic, and nuclear extracts were electrophoresed on a 4–12% polyacrylamide gel (Invitrogen) and proteins were transferred to an Immobilon membrane (Millipore) and blotted with antibodies (Table 2). Blots were imaged using the LiCor Odyssey imaging system and densitometric analysis was performed using ImageStudio (LiCor). In the figures, cropped images grouped together are from the same gel. No images have been spliced together and no images from separate blots have been grouped together. Full images of all blots are available in the Supplementary Information File.

**Table 2:** List of antibodies for Western Blot analysis.

Antibody	Vendor	Catalog Number
Anti-IKKA	Cell Signaling Technology	2682
Anti-IKKB	Cell Signaling Technology	2370
Anti-NFKB P105/P50	Abcam	ab32360
Anti-C-REL	Cell Signaling Technology	12707
Anti-TLR4	Santa Cruz Biotechnology	sc-293072
Anti-MYD88	Abcam	ab2064
Anti-P65	Cell Signaling Technology	8242
Anti-GAPDH	Cell Signaling Technology	5174
Anti-IKBA	Cell Signaling Technology	4814
Anti-IKBB	R&D Systems	AF5225
Anti-P105	Cell Signaling Technology	4717
Anti-Calnexin	Enzo Life Sciences	ADI-SPA-860-D
Anti-HDAC1	Cell Signaling Technology	5356

### Statistical analysis

For comparison between treatment groups, the null hypothesis that no difference existed between treatment means were tested by Student’s *t*-test for two groups and two-way ANOVA for multiple groups with potentially interacting variables (sex, duration of exposure), with statistical significance between and within groups determined by means of Bonferroni method of multiple comparisons (Prism, GraphPad Software, Inc). Statistical significance was defined as p < 0.05.

## Results

### LPS-induced disruption of lung development is similar in male and female mice

**Figure 1:**
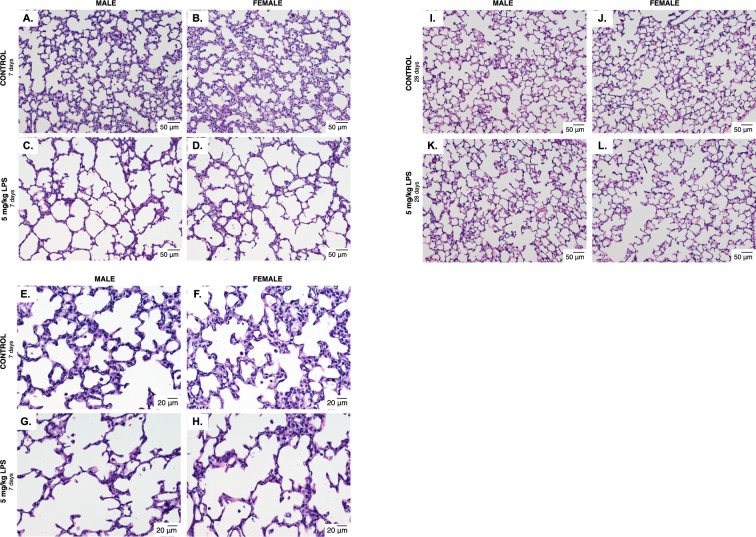
Early postnatal systemic LPS exposure impairs lung development in male and female mice. Representative hematoxylin and eosin stained photomicrographs of lung tissue from 7 day old (**A**) male and (**B**) female control mice, and 7 day old **(C)** male and **(D)** female mice exposed to systemic LPS (5 mg/kg, IP, P0). All images were obtained using the 20x objective lens, internal scale bar 50uM. **(E)** male and **(F)** female control mice, and 7 day old **(G)** male and **(H)** female mice exposed to systemic LPS (5 mg/kg, IP, P0). All images were obtained using the 40x objective lens, internal scale bar 20uM. Representative hematoxylin and eosin stained photomicrographs of lung tissue from 28 day old **(I)** male and **(J)** female control mice, and 28 day old **(K)** male and **(L)** female mice exposed to systemic LPS (5 mg/kg, IP, P0). All images were obtained using the 20x objective lens, internal scale bar 50uM.

Perinatal systemic LPS exposure results in lung injury and abnormal development^17–31^. To assess whether the effect of LPS on the developing lung was sex specific, we exposed neonatal (P0) male and female mice to systemic LPS (5 mg/kg, IP). Pups were allowed to recover, and we then assessed lung development on P7 and P28. Both male (Fig. 1A,E) and female (Fig. 1B,F) control mice demonstrated normal and similar lung development at P7 and P28. We found that LPS disrupted lung development of both male (Fig. 1C,G) and female (Fig. 1D,H) neonatal mice, visibly marked by simplified alveolar structure and enlarged airspaces. By P28, LPS-induced abnormalities in lung structure noted at P7 appeared to have attenuated in male and female mice (Fig. 1I–L).

**Figure 2:**
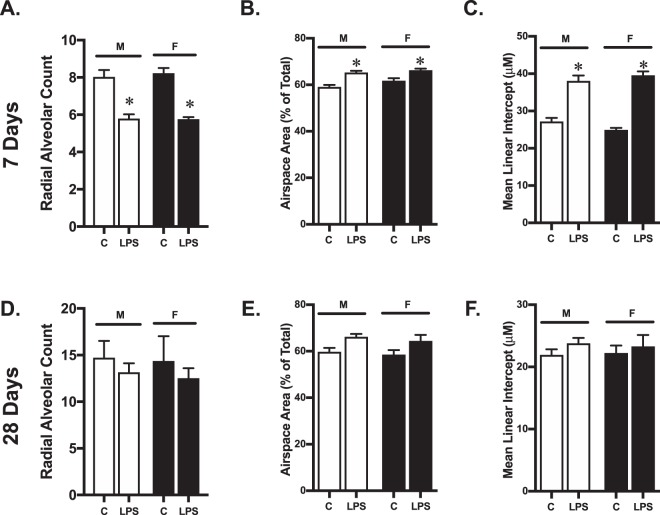
Impaired lung development induced by early postnatal systemic LPS exposure does not differ between male and female mice. **(A)** Radial alveolar counts, **(B)** airspace area **(C)** and mean linear intercept at 7 days of life in male and female control mice (**C**) or following exposure to systemic LPS (5 mg/kg, IP, P0). **(D)** Radial alveolar counts, **(E)** airspace area **(F)** and mean linear intercept at 28 days of life in male and female control mice (**C**) or following exposure to systemic LPS (5 mg/kg, IP, P0). Values are means ± SE from 6 individual animals per sex per condition. *p < 0.05 vs. sex-matched unexposed control.

Objective measures of lung development, including RAC (Fig. 2A), airspace area (Fig. 2B), and MLI (Fig. 2C) were not different between control male and female mice at P7. Systemic LPS exposure resulted in decreased RAC (Fig. 2A), increased airspace area (Fig. 2B) and increased MLI (Fig. 2D) in both male and female mice. The degree of injury was similar between male and female mice. Objective measures of lung development, including RAC (Fig. 2D), airspace area (Fig. 2E), and MLI (Fig. 2F) were not different between control male and female mice at P28. The noted differences in lung structure observed at P7 in LPS-exposed male and female mice had attenuated by P28. While LPS-exposed male and female mice showed the similar patterns of changes in RAC (Fig. 2D), airspace area (Fig. 2E) and MLI (Fig. 2F) at P28, these differences were no longer statistically different from controls. Furthermore, no differences were noted between LPS-exposed male and female mice at this time point. These data demonstrate that in ICR mice, abnormal lung development induced by post-natal systemic LPS exposure is not sex-specific, and the resolution of this injury is similar between male and female mice.

### LPS-induced pulmonary and hepatic expression of pro-inflammatory cytokines is similar in male and female mice

**Figure 3:**
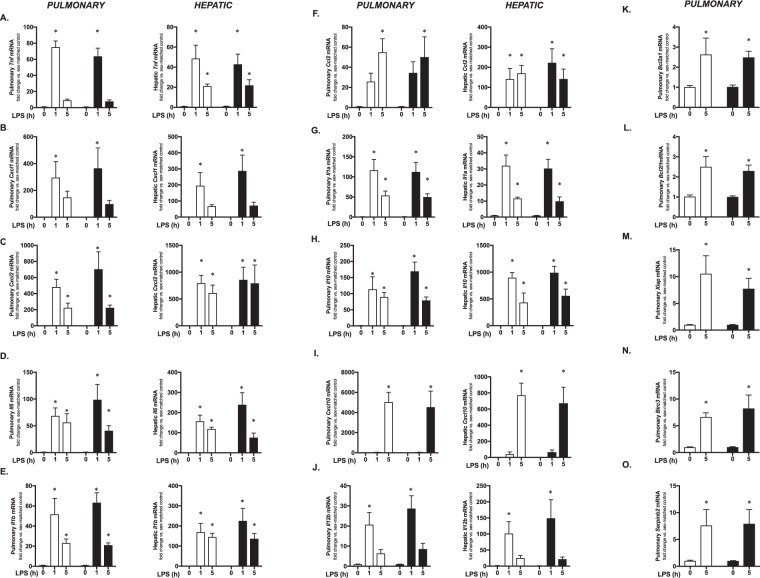
Systemic LPS-induced pulmonary and hepatic gene expression is not different between neonatal male and female mice. Fold induction of pulmonary and hepatic expression of **(A)** *Tnf*, **(B)** *Cxcl1*, **(C)** *Cxcl2*, **(D)** *Il6*, **(E)** *Il1b* and **(F)** *Ccl2*, **(G)** *Il1a*, **(H)** *Il10*, **(I)** *Cxcl10*, **(J)** *Il12b*, **(K)** *Bcl2a1*, **(L)** *Bcl2l1*, **(M)** *Xiap*, **(N)** *Birc3*, **(O)** *Serpinb2*, in neonatal (P0) male (white bars) and female (black bars) following exposure to systemic LPS (5 mg/kg IP, 1–5 hours). Genes were selected based on previous reports linking them to LPS-induced lung injury or sex-specific differences in expression (see Table 1). All values are normalized to sex-specific control mice. Values are means ± SE from 6 individual animals per sex per condition. *p < 0.05 vs. sex-matched unexposed control.

A number of different factors have been evaluated for sex-specific differences following neonatal hyperoxia exposure, implicated in TLR4-mediated lung injury^26,31,40–46^, or have been demonstrated to have sex-specific differences in LPS-induced expression in adult models^47–49^ (Table 3). Furthermore, although LPS injures the developing lung, the liver is central to the innate immune response to endotoxemia^50–64^. Thus, we compared the systemic LPS-induced hepatic and pulmonary expression of multiple innate immune regulators between male and female mice at 1 and 5 hours of LPS exposure (Fig. 3A–J). While LPS exposure significantly increased the expression of all genes tested, none were different between male and female mice.

**Table 3:** References used to identify innate immune target genes implicated in TLR4 mediated lung injury and sex differences.

*Gene Name*	Model Evaluated - Reference		
	Hyperoxia-induced neonatal lung injury	TLR4-mediated developing lung injury	Sex difference in adult models following LPS exposure
*Tnf*	11	27, 31	47, 48
*Cxcl1*	11	40	
*Cxcl2*	11	31	
*Il1b*	11	27, 41, 42, 43, 44	48
*Il6*	11		47, 48
*Ccl2*	11	26,31	
*Il1a*		45	48
*Il10*			47
*Cxcl10*		31, 46	
*Il12b*			49

Additionally, the expression of anti-apoptotic factors has previously been identified as a key mediator or neonatal lung injury^13,16,43^. Therefore, we tested whether there was sex-specific pulmonary expression of antiapoptotic factors previously demonstrated respond to systemic LPS exposure in the neonatal period^43^. We did not test these genes at the early time point (1 hour) as we reasoned that detecting any differences in the induction of the anti-apoptotic program if present would occur at later hours of exposure. We found no difference in the induction of pulmonary expression of *Bcl2a1* (Fig. 3K), *Bcl2l1* (Bcl-XL, Fig. 3L), *Xiap* (Fig. 3M), *Birc3* (Fig. 3N) or *Serpinb2* (PAI-2, Fig. 3O) at 5 hours of LPS-exposure in male and female mice. Thus, we found that exposure to systemic LPS induced expression of all genes tested, but this expression did not differ between male and female mice. These data are consistent with a similar degree of LPS-induced lung injury observed between male and female neonatal mice.

### Baseline pulmonary expression of key TLR4 signaling proteins does not differ between male and female neonatal mice

**Figure 4:**
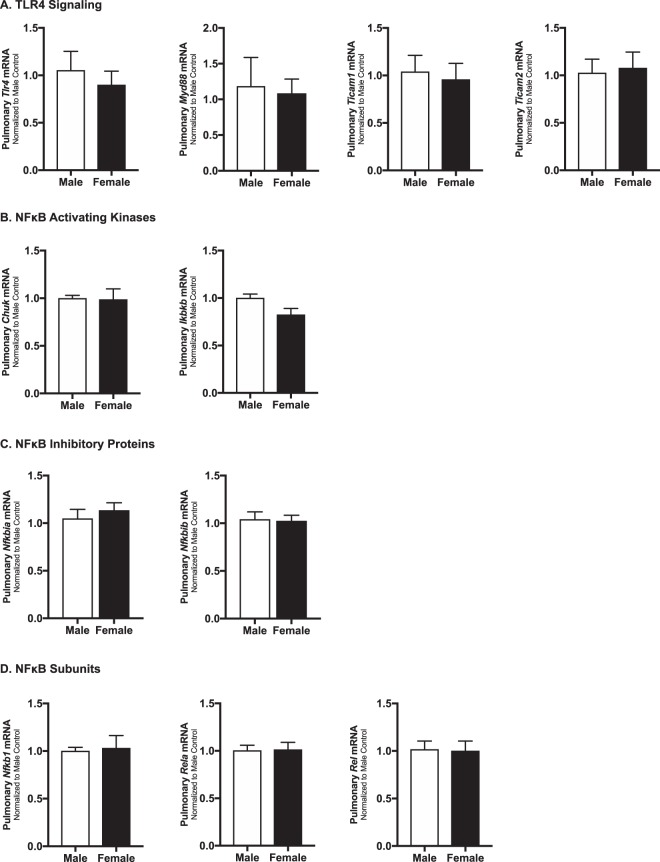
Baseline pulmonary mRNA expression of key components of TLR4 signaling is not different between neonatal male and female mice. Neonatal (P0) male (white bars) and female (black bars) baseline pulmonary expression of **(A)** TLR4 signaling: *TLR4, Myd88, Ticam1, and Ticam2* **(B)** NFκB activating kinases: *Chuk, Ikbkb* **(C)** NFκB inhibitory proteins: *Nfkbia, Nfkbib* **(D)** NF-κB subunits: *Nfkb1, Rela, and Rel*. All values are normalized to male mice. Values are means ± SEM from 6 individual animals per sex.

Previous reports have demonstrated that there are no sex differences in TLR4 expression in the adult rat lung^65^, or in murine macrophage^66^. However, TLR4 expression in the lung is developmentally regulated^67^, and neonatal TLR4 expression is sex specific in other organs^68^. Furthermore, whether pulmonary expression of key mediators of TLR4 signaling is sex-specific is unknown. Thus, we evaluated neonatal pulmonary expression of key mediators of TLR4 signaling (Fig. 4A: *TLR4, Myd88, Ticam1, Ticam2*), NFκB activating kinases (Fig. 4B: *Chuk, Ikbkb*), NFkB inhibitory proteins (Fig. 4C: *Nfkbia, Nfkbib*), and NFκB subunits (Fig. 4D: *Nfkb1, Rela, Rel*) in the absence of LPS exposure. We found no differences in the pulmonary expression of these key components of TLR4 signaling between male and female mice.

**Figure 5:**
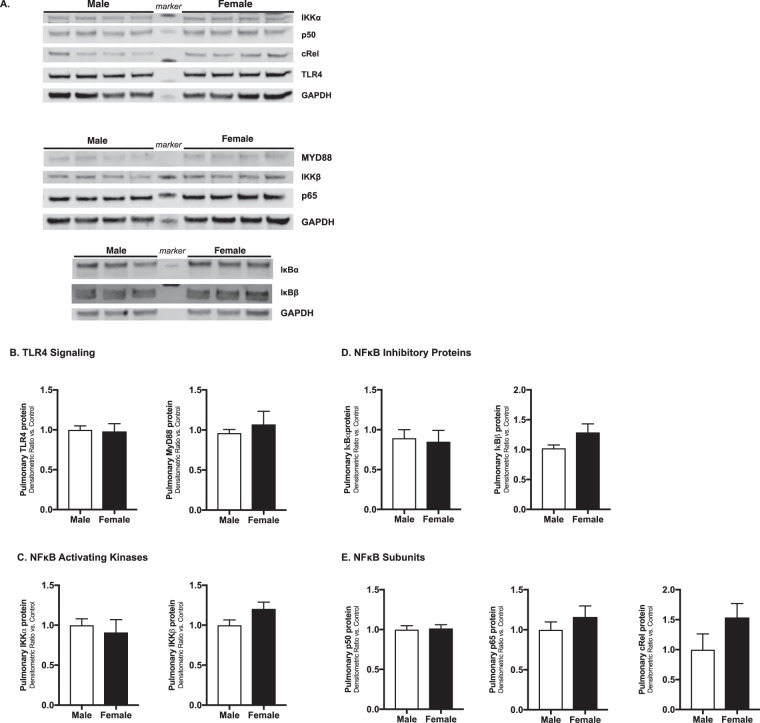
Baseline pulmonary protein expression of key components of TLR4 signaling is not different between neonatal male and female mice. **(A)** Representative Western blots and (**B**) densitometric analysis showing male (white bars) and female (black bars) neonatal (P0) pulmonary expression of **(A**,**B)** key components of TLR4 signaling: TLR4 and Myd88; **(A**,**C)** NFκB activating kinases: IKKα and IKKβ; **(A**,**D)** NFκB inhibitory proteins: IκBα, IκBβ; and **(A**,**E)** NFκB subunits: p50, p65 and cRel. GAPDH as loading control. Values first normalized to GAPDH, and then to male control. Values shown as means ± SEM from 6 individual animals per sex.

Furthermore, we assessed pulmonary protein expression of key components of TLR4 signaling (Fig. 5A,B: TLR4 and Myd88), NFκB activating kinases (Fig. 5A,C: IKKα and IKKβ), NFκB inhibitory proteins (Fig. 5A,D: IκBα, IκBβ), and NFκB subunits (Fig. 5A,E: p50, p65 and cRel) in the absence of LPS exposure. We found no differences in the pulmonary expression of these key components of TLR4 signaling between male and female mice. These data demonstrate that at baseline, prior to any LPS exposure, pulmonary expression of these key mediators of LPS-induced TLR4 signaling is not different between male and female mice.

### Cytosolic pulmonary activation of canonical NFκB signaling induced by systemic LPS exposure does not differ between male and female neonatal mice

**Figure 6:**
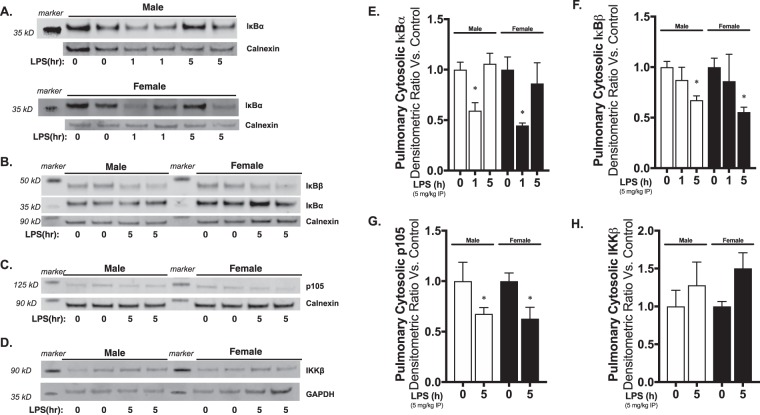
Pulmonary cytosolic IκB degradation in response to systemic LPS is not different between neonatal male and female mice. Representative Western blots of pulmonary cytosolic extracts showing **(A**,**B)** IκBα and IκBβ, **(C)** p105 and **(D)** IKKβ in neonatal (P0) male and female mice following exposure to systemic LPS (5 mg/kg IP, 1–5 hours). Calnexin as loading control. Densitometric analysis of **(E)** IκBα, **(F)** IκBβ, **(G)** p105 and **(F)** IKKβ in pulmonary cytosolic extracts form neonatal male and female mice following exposure to systemic LPS (5 mg/kg IP, 1–5 hours). All values first normalized to loading control, then to unexposed, sex-specific control. Values shown as means ± SEM from 6 individual animals per sex per time point. *p < 0; 0.05 vs. control.

It is well known that canonical NFκB signaling proceeds following TLR4 activation^69^. In order for NFκB complexes to translocate to the nucleus, degradation of the cytosolic NFκB inhibitory proteins IκBα, IκBβ and p105 must occur^33^. Previous studies of sex-specific differences in neonatal lung injury have invoked differences in NFκB signaling^11,12^. Thus, we sought to assess whether the first step penultimate step of NFκB nuclear translocation differed between sexes in the lungs of neonatal mice exposed to systemic LPS. We found similar patterns of IκBα degradation (Fig. 6A,B,E), IκBβ degradation (Fig. 6B,F) and p105 degradation (Fig. 6C,G) in the lungs of male and female neonatal mice exposed to systemic LPS. These data demonstrate similar kinetics of cytosolic TLR4-NFκB signaling in the lungs of neonatal male and female mice exposed to systemic LPS.

Previous studies have implicated differential regulation of the NFκB activating kinase IKKβ in the pathogenesis of sex differences in neonatal hyperoxic lung injury^11,12^. Thus, we assessed pulmonary IKKβ protein expression in the lungs of neonatal male and female mice following exposure to systemic LPS. Consistent with previous reports, we found no differences in baseline IKKβ expression between male and female neonatal mice (Fig. 6D,H)^11^. Furthermore, we found that exposure to systemic LPS did not alter total IKKβ levels in either male or female mice (Fig. 6D,H).

### Nuclear translocation of NFκB subunits induced by systemic LPS exposure does not differ between male and female neonatal mice

**Figure 7:**
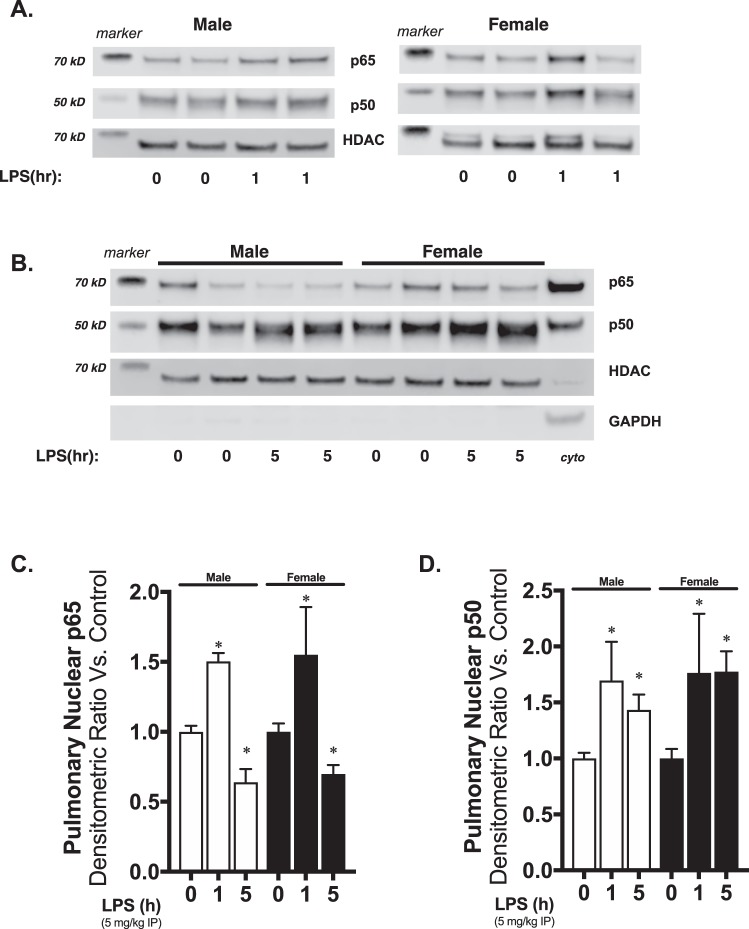
Pulmonary nuclear translocation of the NFκB subunits p65 and p50 in response to systemic LPS is not different between neonatal male and female mice. Representative Western blots of pulmonary nuclear extracts showing **(A)** p65 and p50 in neonatal (P0) male and female mice following 1 hours of systemic LPS exposure (5 mg/kg IP), and **(B)** p65 and p50 following 5 hours of systemic LPS exposure (5 mg/kg IP). Cyto = cytosolic positive control. HDAC as nuclear loading control. GAPDH shown as evidence of purity of nuclear extract. Densitometric analysis of **(C)** p65, **(D)** p50 in pulmonary nuclear extracts form neonatal (P0) male and female mice following exposure to systemic LPS. All values first normalized to loading control, then to unexposed, sex-specific control. Values shown as means ± SEM from 6 individual animals per sex per time point. *p < 0; 0.05 vs. control.

In our final comparison of LPS-induced pulmonary TLR4 signaling in neonatal male and female mice, we assessed nuclear translocation of the key NFκB subunits p65 and p50. At early time points, we found significantly elevated nuclear p50 and p65 in both male and female LPS-exposed mice (Fig. 7A,C,D). At later time points, p65 levels were significantly lower than baseline, while p50 remained elevated in both male and female mice (Fig. 7B–D). Of note, previous studies have shown that NFκB activity and nuclear translocation of NFκB is oscillatory following innate immune stimulation^70–76^. Thus, the observed dynamic changes in nuclear NFκB subunits demonstrates that oscillatory NFκB activity is similar between LPS-exposed male and female mice. Thus, the similarities between male and female mice demonstrate the absence of difference in pulmonary TLR4 signaling and subsequent NFκB activation following exposure to systemic LPS.

## Discussion

We found that in response to early postnatal systemic LPS challenge, there was no difference in pulmonary injury and abnormal lung development between male and female mice. Following systemic LPS exposure on P0 mice, both males and females demonstrated evidence of abnormal lung development with decreased radial alveolar counts, increased airspace area and increased mean linear intercept. The deviations from control were similar in LPS-exposed male and female mice, indicating a lack of sex difference in terms of lung injury in response to systemic LPS exposure. Because previous reports have demonstrated sex differences in hyperoxia-induced neonatal lung injury and abnormal development, we sought to assess pulmonary expression of factors previously implicated in sex differences and lung injury, as well as the key components of TLR4-NFκB signaling in male and female mice. We found no differences in the pulmonary expression of key mediators of lung injury or apoptosis between LPS-exposed male and female neonatal mice. Furthermore, we found no difference between male and female mice in the baseline pulmonary expression of key mediators of TLR4-NFκB signaling, or in the kinetics of LPS-induced NFκB activation as assessed by cytosolic IκB inhibitory degradation and nuclear translocation of activating NFκB subunits. Together, these phenotypic, transcriptional, and mechanistic data support the conclusion that the innate immune response to early postnatal LPS exposure and resulting pulmonary sequelae are similar in male and female mice.

These results are interesting because clinical studies have reported sex-specific pulmonary implications following preterm delivery. While an increased risk of developing bronchopulmonary dysplasia (BPD) has been reported in males^2–4^, an increased incidence in persistent lung function following exposure to chorioamnionitis has been reported in in former preterm female neonates^77^. Importantly, not all studies agree that males have worse long-term respiratory morbidities following preterm birth^78^. These results argue for a better understanding of the mechanisms underlying the pulmonary response to various oxidant and inflammatory stressors encountered in the neonatal period and following preterm delivery.

Various pre-clinical models of neonatal lung injury exist^6,9^. Neonatal hyperoxia exposure is a well-established model that induces consistent and significant lung injury. Importantly, Lingappan and colleagues demonstrated that neonatal hyperoxia-induced lung injury was worse in male compared to female mice^11^. Of note, hyperoxic exposure was associated with evidence of increased NFκB activation in female mice, with increased levels of the active phosphorylated p65 in females and lower levels of the activating kinase IKKβ in males. These findings are consistent with previous reports demonstrating a protective effect of pulmonary NFκB signaling following hyperoxia exposure^13,14,16^.

Exposing the canalicular and saccular stage of lung to LPS consistently results in inflammation, injury and abnormal development across multiple species^17–31^. Similar to hyperoxia-induced pulmonary NFκB activity, LPS-induced NFκB activity plays some protective role in the neonatal lung^31^. While NFκB controls the expression of multiple likely injurious pro-inflammatory cytokines and chemokines, complete inhibition of LPS-induced NFκB activation exacerbates neonatal lung injury^31^. However, while pulmonary NFκB signaling has been implicated in the attenuated injury observed in female neonatal mice following hyperoxia exposure^11,12,79^, these results may not apply to lung injury and subsequent abnormal development following exposure to LPS. Importantly, NFκB activity following exposure to inflammatory stimuli results from signaling events that are likely independent from those leading to activation following exposure to oxidant stress. With exposure to inflammatory stress (eg. LPS-induced TLR4 activation), the IκB inhibitory proteins are phosphorylated and degraded, allowing NFκB nuclear translocation and DNA binding^32,33^. While the NFκB activation cascade occurring after exposure to inflammatory stress is well defined, the definitive pathway that occurs following exposure to oxidant stress remains debatable^34–37^. Therefore, the well-characterized sex-type specific differences in response to hyperoxia in the neonatal period may not be applicable to injury following exposure to inflammatory stress.

Previous studies have demonstrated that there are sex-specific differences in the innate immune response that are dynamic over the life course^80–82^. Multiple studies have interrogated whether there are sex-specific differences in TLR4 innate immune signaling in adults. The results of these studies are decidedly mixed, and these mixed results are likely related to differences in study design, dose of LPS used, the model organism used, the maturational stage of the organism, and whether the exposure was performed on cells in culture or *in vivo*. Proinflammatory cytokine (TNFα, IL-1β) levels were higher in LPS-exposed whole blood, neutrophils, and peripheral blood mononuclear cells obtained from healthy human male volunteers compared to female^83–86^, and LPS-induced IL-1β expression was higher in macrophages isolated from adult male mice compared to female^66^. In contrast to these findings, some studies have demonstrated a more robust response to LPS in adult female rodents and cells derived from adult females when compared to males. For example, in response to IV LPS challenge, adult female human volunteers demonstrate a more robust proinflammatory response compared to males^87,88^. Similarly, pro-inflammatory cytokine expression is higher in LPS-exposed adult female mice^89^ and macrophages isolated from adult female mice^90^ compared to males. Finally, it has even been published that there are no differences in circulating TNFα and IL-1β in male and female adult mice following IP LPS challenge^91^.

However, whether any of these findings guide our understanding of neonatal period is unclear. Significant progress has been made in determining differences between the neonatal and adult response to LPS challenge and TLR4 stimulation. While important differences between adults and neonates have been identified, most of the studies comparing early life TLR4 responsiveness have not evaluated for sex differences^92–100^. Furthermore, in the scant data that are available, reports are not consistent. Levels of LPS-stimulated cord blood IL-1β and IL-6 secretion are higher in males compared to females^101^. In contrast to this finding, no difference in LPS-stimulated cytokine levels were noted from LPS-exposed monocytes obtained from cord blood of preterm male and female neonates^102^. Following early neonatal IP LPS challenge in rats, systemic cytokine levels were not different between males and females, although mortality was higher in males^103^. Our data adds to this literature, and supports the conclusion that in response to early neonatal systemic LPS challenge, the acute innate immune response and early pulmonary sequelae in male and female mice are more similar than they are dissimilar.

Given our growing appreciation of the differences between male and female innate immunity, it is important to question our results and query the existing literature to better understand these findings. Given the known influence of sex hormones on the innate immune response^82^, studies have been done examining sex-specific differences in pre- and post-pubertal mice. Following IP LPS challenge, circulating levels of TNFα, IL-6, and IL-10 were not different between pre-pubertal male and female mice^47^. In contrast, post-pubertal mice demonstrated a sex difference, with circulating levels being higher in males compared to females^47^. The influence of sex hormones is further highlighted by the finding that pre-pubertal female mice are resistant to LPS-induced mortality comparted to post-pubertal females^104^. Human data support these findings, as levels of IL-1β, IL-6, and TNFα were not different between LPS-exposed male and female whole blood samples^105^. Of note, macrophage TLR4 expression is responsive to sex hormone levels^106,107^. In the current study, we did not assess our mice for sex hormone levels. However, previous studies have shown that there is a testosterone surge in male mice shortly after delivery, with levels returning to baseline and no longer different from females by 4–6 hours of life^108,109^. Furthermore, serum estrogen levels are similar in male and female neonatal rats^110^. Thus, it is possible that the confluence of the developing innate immune system and relatively similar levels of sex hormones present in the early neonatal period obviate the sex differences reported with post-pubertal LPS challenge.

There are important limitations to the current study. Here, we used systemic LPS challenge to stimulate the TLR4 mediated innate immune response. There are limitations to using endotoxemia as a clinically relevant model^111^. Namely, LPS challenge is a single exposure to a sterile stimulus. While it is very useful to interrogate TLR signaling, because there is no ongoing bacterial presence as there is with an active infection, the conclusions that can be drawn are limited. Thus, while these studies represent a first step, more work must be done using more complex models to interrogate sex-differences in the innate immune response. Additionally, we assessed the transcriptional response at 1 and 5 hours after exposure to systemic LPS. It is quite likely that significant changes occur before the one-hour time point, and beyond the 5 hour time point. One important remaining area to investigate is sex-differences in the factors controlling resolution of inflammation^112^. Importantly, we did not assess any markers of the mechanisms responsible for the resolution of inflammation. It is possible that while male and female neonatal mice have similar acute responses to LPS-induced TLR4 signaling, the time course to resolution and the factors controlling that process are different. How these mechanisms may affect the developing lung, and whether this occurs in a sex-specific manner, is unknown. Along these lines, we assessed lung morphometrics at 7 and 28 days following a single, early postnatal innate immune stimulus. While lungs of both LPS-exposed male and female mice are abnormal at postnatal day 7, it appears that the lungs are recovering by the 28 day time point. At this point, the abnormalities noted in RAC, airspace area, and mean linear intercepts at P7 in LPS-exposed male and female mice have attenuated. Thus, while the lung is recovering, it is not known if the mechanisms underlying that recovery are sex specific, or how the recovering lung would respond to a second injurious exposure. Additionally, it is unknown whether there are sex-specific differences in lung function, as only morphometrics were assessed here.

In conclusion, we found that following early postnatal systemic LPS challenge, both male and female neonatal mice demonstrated evidence of pulmonary injury and abnormal lung development. Objective markers showed that lung development was similarly impaired in LPS-exposed male and female mice. We interpret these data to support a lack of sex difference in terms of lung injury in response to systemic LPS exposure. Importantly, male and female pulmonary expression of key member of TLR4-NFκB signaling cascade was similar, as was the LPS-induced expression of pro-inflammatory and apoptotic mediators of lung injury. Lastly, the kinetics of LPS-induced pulmonary TLR4-NFκB signaling was similar in male and female neonatal mice. We speculate that in the early postnatal period, the pulmonary innate immune response to TLR4 stimulation is more similar than dissimilar in male and female mice. These results have implications for treatment strategies aimed at attenuating lung injury and abnormal development following exposure to inflammatory stress following preterm delivery.

## Supplementary information

Full Blot Images
