# Supplementary material for: Sex-differences in LPS-induced neonatal lung injury
Source: Sci Rep. 2019 Jun 11;9:8514. doi: 10.1038/s41598-019-44955-0 (PMC6560218; doi:10.1038/s41598-019-44955-0)

Sex-differences in LPS-induced neonatal lung injury

Leanna Nguyen, Odalis Castro, Robyn De Dios, Jeryl Sandoval, Sarah McKenna, Clyde J. Wright

TOP IMAGES in Figure 5 A, presented in the order of antibody exposure

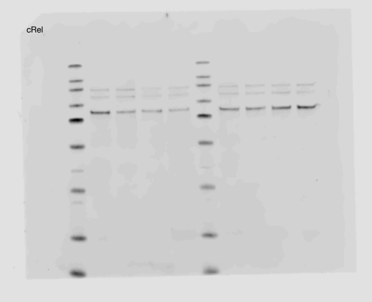

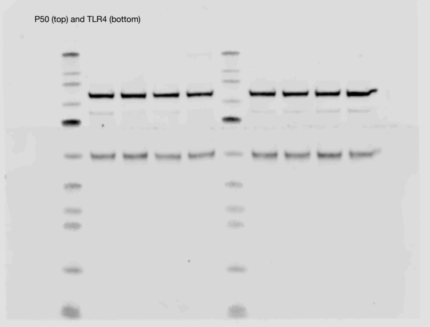

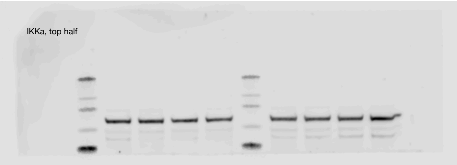

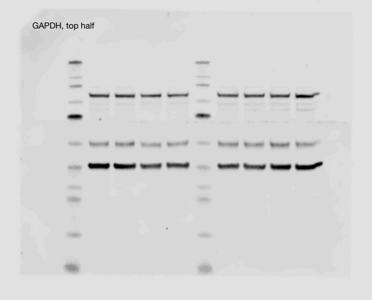

Middle IMAGES in Figure 5 A, presented in the order of antibody exposure

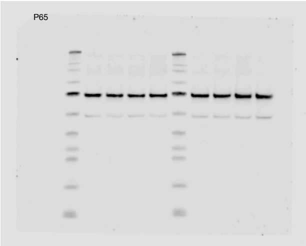

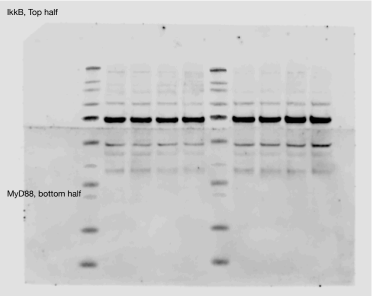

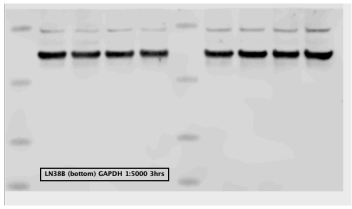

Bottom IMAGES in Figure 5 A, presented in the order of antibody exposure

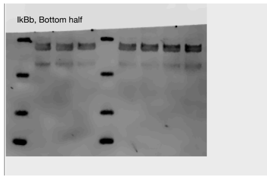

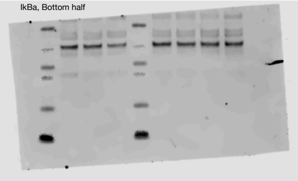

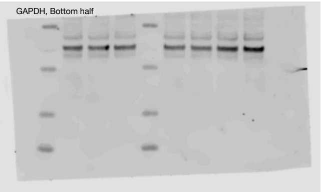

Figure 6A

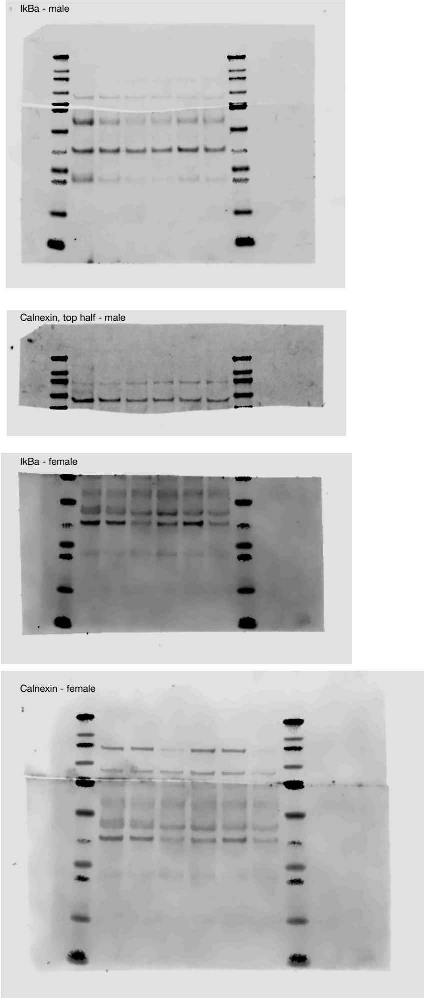

Figure 6B

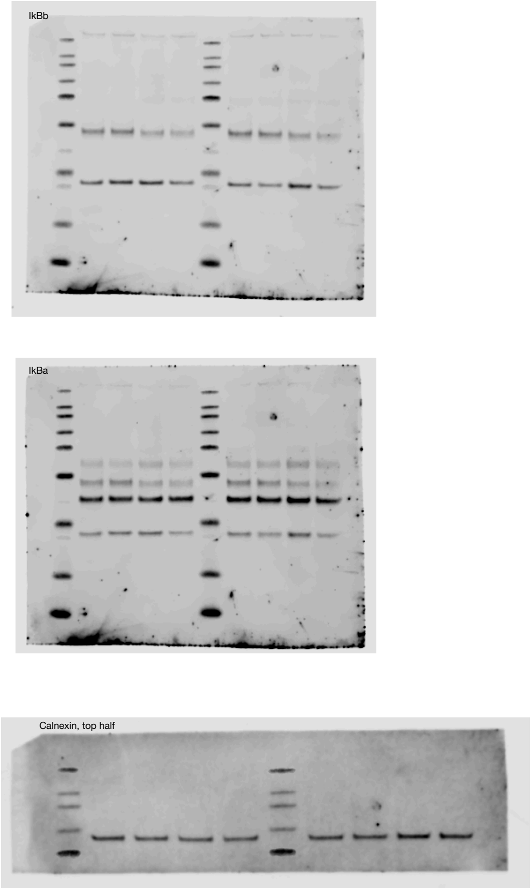

Figure 6C

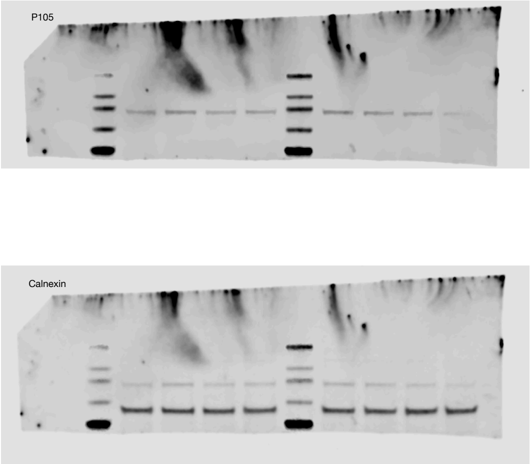

Figure 6d

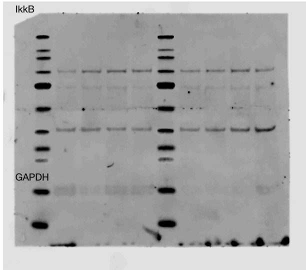

Figure 7A

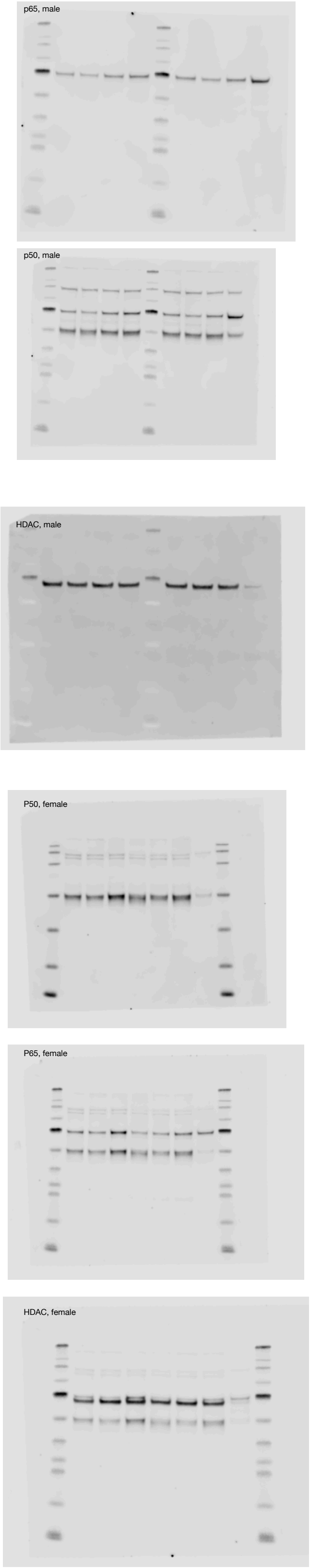

Figure 7B

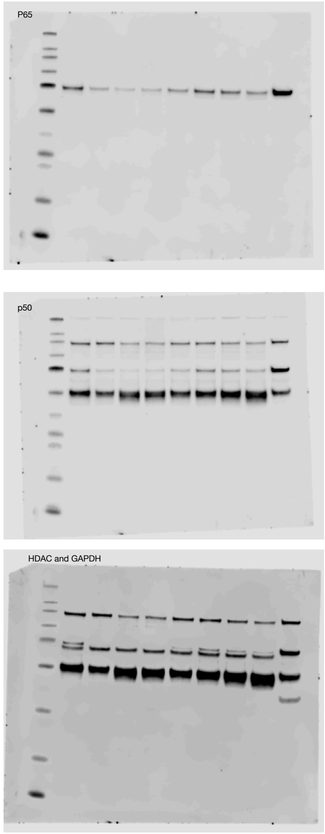

Supplement: Supplementary file 1 — Full Blot Images [file 41598_2019_44955_MOESM1_ESM.pdf]
